# From the Editor’s Desk

**Published:** 2015

**Authors:** Pat Commerford

**Affiliations:** Editor-in-Chief

A patient presenting with a large pericardial effusion of uncertain aetiology is a relatively common clinical problem facing practitioners in Africa. The optimal management of such patients, particularly in resource-constrained environments, remains unclear. Tuberculosis is generally considered to be the most important likely cause, particularly if numerous fibrin strands are seen to be present on echocardiography, and many practitioners would advocate the immediate institution of treatment for this disease under these circumstances. The issue is far from clear however.

Treatment for tuberculosis, in addition to its obvious benefits, carries some risk of serious adverse events but it also has significant ‘minor’ side effects that are thoroughly unpleasant for patients. In addition, there is the risk of missing other treatable causes of such an effusion. While acknowledging that such a risk is real, many would argue it is relatively unimportant, given the fact that most alternative diagnoses are neither treatable nor curable.

The counter-argument is that detailed and comprehensive investigation of all pericardial effusions may enhance diagnostic certainty and identify the few patients who may benefit from alternative treatment. The problem is that detailed investigations, including pericardiocentesis, advanced imaging studies or open biopsy carry their own risks, are often not available and if they are, the results of such investigations are largely untested in terms of diagnostic sensitivity and specificity. The case report of Tembani-Munyandu and colleagues (online, page e7) serves to highlight this problem.

There is a great need for a properly structured, adequately powered study to evaluate the risks and benefits of instituting empirical therapy for tuberculosis, compared to aspiration and detailed investigation in patients with large pericardial effusions in Africa. Such a study would be complex, needing to recruit a range of patients, including persons both HIV positive and negative, but given the scope of the problem and the importance of the question being addressed, it is essential that it be conducted.

The effects of liver disease on cardiac structure and function have been argued and discussed for decades with varied outcomes. Bekler and colleagues (page 110) demonstrated that patients with non-alcoholic fatty liver disease (NALFD) had right ventricular diastolic dysfunction. It is not clear whether the authors considered that these abnormalities of cardiac function were directly related to the NALFD or to accompanying components of the metabolic syndrome. Any therapeutic intervention that may be contemplated will require clarity in this regard.

In hospital-based practice, it is alarming to see just how often QT prolongation on the ECG is ignored, missed and/or underreported, and knowledge of the hazards of administering agents prolonging the QT interval and situations where such agents may be harmful is often limited. The case report of McCuthcheon and Manga (page 145) serves to draw attention to the use of commonly used drugs, in this case erythromycin, that can cause lethal arrhythmias in predisposed individuals when they are used inappropriately.

Sahin and colleagues (page 121) report on the frequency of atrial fibrillation after surgery for left atrial myxoma. Not surprisingly, age proved to be one of the predictors of atrial fibrillation in this situation, as it does in all others. The mechanism/s of the powerful effects of age on the development of atrial fibrillation are complex and not fully explained but an age-associated relationship with the development of atrial fibrillation is not unexpected.

